# Memory Clinics and Day Care Centers in Thessaloniki, Northern Greece: 30 Years of Clinical Practice and Experience

**DOI:** 10.3389/fneur.2021.683131

**Published:** 2021-08-25

**Authors:** Magda Tsolaki, Marianna Tsatali, Mara Gkioka, Eleni Poptsi, Anthoula Tsolaki, Vasileios Papaliagkas, Irene-Maria Tabakis, Ioulietta Lazarou, Marina Makri, Dimitrios Kazis, Sotirios Papagiannopoulos, Andreas Kiryttopoulos, Efrosyni Koutsouraki, Thomas Tegos

**Affiliations:** ^1^Greek Association of Alzheimer's Disease and Related Disorders (GAADRD), Thessaloniki, Greece; ^2^1st University Department of Neurology UH “AHEPA”, School of Medicine, Faculty of Health Sciences, Aristotle University of Thessaloniki, Thessaloniki, Greece; ^3^Laboratory of Neurodegenerative Diseases, Center for Interdisciplinary Research and Innovation (CIRI - AUTh) Balkan Center, Buildings A & B, Aristotle University of Thessaloniki, Thessaloniki, Greece; ^4^3rd University Department of Neurology “G. Papanikolaou” Hospital, Faculty of Health Sciences, School of Medicine, Aristotle University of Thessaloniki, Thessaloniki, Greece; ^5^Department of Biomedical Sciences International Hellenic University, Thessaloniki, Greece

**Keywords:** memory, dementia, day care centers, educational programs, Alzheimer association, neurology departments, non-pharmacological interventions

## Abstract

**Background:** This review describes the diagnostic and interventional procedures conducted in two university memory clinics (established network of G. Papanikolaou Hospital: 1988–2017 and AHEPA hospital: 2017–today) and 2 day care centers (established network of DCCs: 2005–today) in North Greece and their contribution in the scientific field of dementia. The aims of this work are (1) to provide a diagnosis and treatment protocol established in the network of memory clinics and DCCs and (2) to present further research conducted in the aforementioned network during the last 30 years of clinical practice.

**Methods:** The guidelines to set a protocol demand a series of actions as follows: (1) set the diagnosis criteria, neuropsychological assessment, laboratory examinations, and examination of neurophysiological, neuroimaging, cerebrospinal fluid, blood, and genetic markers; and (2) apply non-pharmacological interventions according to the needs and specialized psychosocial interventions of the patient to the caregivers of the patient.

**Results:** In addition to the guidelines followed in memory clinics at the 1st and 3rd Department of Neurology and two DCCs, a database of patients, educational programs, and further participation in international research programs, including clinical trials, make our contribution in the dementia field strong.

**Conclusion:** In the current paper, we provide useful guidelines on how major and minor neurocognitive disorders are being treated in Thessaloniki, Greece, describing successful practices which have been adapted in the last 30 years.

## Introduction

Dementia has been described as a clinical syndrome caused by neurodegeneration (Alzheimer's disease, Lewy body, and frontotemporal dementia being the most common pathologies) or as a secondary syndrome (vascular, metabolic, hormonal, and infectious dementia), characterized by progressive deterioration in cognitive ability, behavior, and capacity for independent living (1). Typically, it is a condition that usually affects older people (2, 3). Because of a longer life expectancy along with the lack of efficient therapeutic strategies, dementia is increasingly becoming a major public health problem. According to Alzheimer Disease International, it has been estimated that 35.6 million people were living with dementia worldwide in 2010, with the numbers expected to almost double every 20 years up to 65.7 million in 2030 (1). In Greece, there are almost 196,000 people living with dementia, while in 2050 this number is going to increase to 356,000. Moreover, family caregivers are estimated at 400,000 all over the country. Few studies have been conducted so far concerning the prevalence of dementia and mild cognitive impairment (MCI) in Greece (4–7), but the latest data revealed that the overall prevalence of dementia is 5.0%, with 75.3% of the cases attributed to Alzheimer's disease (8).

Thessaloniki, located in northern Greece, is the second biggest city of the country with high contribution in dementia research and clinical practice. The memory and dementia network in Thessaloniki, which started with the so-called Outpatient Memory and Dementia Clinic (3rd Department of Neurology), which was established in 1988 at “G. Papanikolaou” General Hospital (established network 1988–2017). Years later and specifically in 1995, Professor Magda Tsolaki, with the cooperation of dementia experts, founded the Association of Alzheimer's Disease and Related Disorders (GAADRD) which is responsible for 2 day care centers (DCCs) in Thessaloniki. Since 2005, the team of experts had the opportunity to expand the network and establish in total four DCCs in several cities across Greece (Thessaloniki, Volos, Chania, and Athens). At the end of 2017, the memory and dementia network was established to the Outpatient Memory and Dementia clinic (1st Department of Neurology) at “AHEPA” University Hospital till today. The aforementioned network between memory clinics and DCCs offer medical treatment, psychological support, and non-pharmaceutical interventions to beneficiaries who range from no cognitive impairment (NCI), subjective cognitive impairment (SCI), MCI, and dementia. Additionally, many projects and clinical trials are also being implemented with the collaboration of several dementia scientists abroad. Moreover, a large electronic database containing the information of all patients has been developed for clinical purposes. Consequently, the memory and dementia network provides high-quality diagnostic, treatment, and support services to individuals affected by major or minor neurocognitive impairment and their caregivers or family members in North Greece. Given that this initiative constitutes a significant part of global research groups, the memory and dementia network works in line with high standards provided worldwide.

The goals of this work are (1) to provide a diagnosis and treatment protocol established in memory clinics and DCCs and (2) to present further research conducted in the last 30 years of clinical practice.

## Setting

### Memory Clinics

The current memory clinic network includes the outpatient memory clinic of a university general hospital (AHEPA), where the initial diagnosis and follow-up assessments patients as well as education of students, including academic lectures and staff meetings, take place. The outpatient clinic operates once per week under the umbrella of the general hospital and health ministry. It consisted of a neurologist, nurses, medical students, and psychologists offering services of full screening, diagnosis, and medical treatment. Patients who visit the memory clinic, for any reason, follow the screening/diagnostic protocol, and after giving out the results and prescription of medication, they are recommended to visit a DCC for further benefits according to their needs, such as non-pharmaceutical interventions. Moreover, the research and academic team developed a new postgraduate program in 2020 (master's degree) entitled “Neuroscience and Neurodegenerative Diseases,” and therefore professionals who work on the dementia field provide new treatment horizons both in beneficiaries as well as in the research field.

### Alzheimer Hellas DCCs

GAADRD is a non-governmental organization and member of European as well as international organizations such as Alzheimer Europe and Alzheimer Disease International. It consists of neurologists, psychiatrists, general practitioner, psychologists, biologists, social workers, physical trainers, physiotherapists, and nurses who have been specially trained and educated. The DCCs under the umbrella of GAADRD are prototype and perfectly organized centers offering diagnosis and several non-pharmacological programs for the beneficiaries, namely: (a) programs of cognitive training for people with MCI and people with dementia (PwD) of first stages and (b) cognitive stimulation programs for people with mild and moderate stage of dementia. The participants attend cognitive training or stimulation programs for one or several days per week, following a protocol according to their needs, such as cognitive deficits, mood disorders, and functionality problems. The entrance to the group is determined by a psychologist who is an expert in non-pharmaceutical programs. Each program duration is almost a year. Furthermore, there are also prevention programs to minimize the conversion of SCI to MCI and dementia as well as those delivered to NCI healthy older adults who are at risk of developing dementia due to family history or other relevant health problems. Furthermore, psychotherapeutic programs are also provided to caregivers in order to support them during their caregiving role. Additionally, in the last 15 years, 1-h lectures are conducted every week, including the most recent developments in the research of neurodegenerative diseases as well as many educational projects for caregivers all over Greece. Finally, GAADRD has organized 12 national conferences, one Alzheimer Europe Conference (2003), and one Alzheimer Disease Conference (2010). GAADRD also contributed to the national observatory for dementia in Greece (2013) and one Satellite AAIC Athens Conference (2021) and has also organized DCCs all over Greece and Egypt. Since 2001, GAADRD has been a member of the European Alzheimer Disease Consortium (EADC).

## Diagnostic Methods

The diagnostic procedure officially takes place in DCCs or in outpatient memory clinics. All patients who visit the outpatient memory clinics are screened for cognitive deficits with a neuropsychological battery (Tables 1, 2), while laboratory examinations, neurophysiological and neuroimaging examination, and genetic markers are also conducted (Table 3). The memory clinic's services are used as “a hub” of patients diagnosed with a cognitive disorder. Subsequently, some of them, if they need it, are referred to DCCs for further neuropsychological assessments (Table 2) and psychological support and to attend non-pharmaceutical programs. Vice versa, patients who visit a DCC for the first time after diagnosis may visit the memory clinic to undertake specialized examinations.

**Table 1:** Screening tools.

**Screening tools**	**Domain**	**Greek cutoff scores**
Clinical dementia rating (CDR)^a,b^ (9)	Global cognition	Translation and adaptation to Greek (study under preparation)
Global deterioration scale (GDS)^a,b^ (10)	Global cognition	(11)
Mini mental state examination MMSE (MMSE)^a,b^ (12)	Global cognition	(13)
Montreal cognitive assessment (MoCA)^a,b^ (14)	Global cognition	(15)
Hindi mental state examination (HMSE)^a,b^	Global cognition for illiterates	Translation and adaption to Greek (study under preparation) (16)
Alzheimer's disease assessment scale cognitive subscale [ADAS-Cog, (10)]^b^ (17)	Global cognition	(18)
Confusion assessment method (CAM)^a^ (19)	Delirium	Translation and adaption to Greek (study under preparation)
Cognitive decline questionnaire (SCDQ)^a,b^ (20)	SCI	Translation and adaption to Greek (study under preparation)
Memory alternation test (MAT)^a,b^ (21)	SCI	(Lazarou et al. under revision)

a *Tools included in the neuropsychological assessment of memory clinic*.

b *Tools included in the neuropsychological assessment of day care centers*.

**Table 2:** Further neuropsychological assessment.

**Neuropsychological assessment**	**Domain**	**Greek cutoff scores**
Rivermead behavioral memory test (RBMT)^a^ (22)	Memory	(23)
Rey auditory–verbal learning test (RAVLT)^b^ (24)	Verbal learning	(25)
Boston naming test (BNT)^b^ (26)	Language	(27)
Boston diagnostic aphasia examination (BDAE; subtests of narrative writing, repetition, phonemic correlation, and reading comprehension of sentences^b^ (28)	Language	(29)
Verbal fluency test^b^	Language	(30)
Rey figure complex test (copy, immediate, and free delayed recall and recognition trial)^b^ (31)	Visuospatial ability	(32)
Stroop test^b^ (33)	Executive function, processing speed and attention functions	Greek cutoff scores derived from Tsolaki et al. (18), Zalonis et al. (34)
Trail making test A and B (TMT-B)^b^ (35)	Executive function, processing speed and attention functions	(36)
Wechsler adult intelligence scale (WAIS-FSIQ) digit span (forward and backward digit span)^b^ (37)	Short-term memory and working memory	Translation and adaption to Greek (study under preparation)
Digit symbol substitution (DSST)^b^ (37)	Working memory, learning	(38)
Instrumental activities of daily living (IADL)^b^ (39)	Independent living capacity	(40)
Functional rating scale for symptoms of dementia (FRSSD)^a,b^ (41)	Independent living capacity	Translation and adaption to Greek (study under preparation)
Functional cognitive assessment scale (FUCAS)^a,b^ (42)	Independent living capacity	(42)
Neuropsychiatric inventory (NPI)^a,b^ (43)	Behavioral disorders	(44)
Geriatric depression scale (GDS)^a,b^ (45)	Depression	(46)
Short anxiety screening test (SAST)^a,b^ (47)	Anxiety	(48)
Perceived Stress Scale (PSS)^b^ (49)	Anxiety	(50)

a *Tools included in the neuropsychological assessment of memory clinic*.

b *Tools included in the neuropsychological assessment of day care centers*.

**Table 3:** Further examinations (neurochemical and biomarkers).

	**Domain**	**Greek cut off score**	**Cut off scores**
**Laboratory examinations**			
Regular blood test examination^a,b^	Medical view of patient	(51)	
Cerebrospinal fluid examination^a,b^	Identify markers for AD/other dementias, MCI, or healthy	(52)	(52–54)
**Neurophysiological and neuroimaging markers**			
Auditory event-related potentials (AERPs)^a,b^	Identify markers for AD/other dementias, MCI, or healthy	(55, 56)	
Electroencephalography (EEG)^a,b^	Identify markers for AD/other dementias, MCI, or healthy	(57–60)	(54, 61)
HD-EEG recordings (GES 300-256 HCGSN)^b^	Identify markers for AD/other dementias, MCI, or healthy	(62–66)	
MRI^a,b^	Identify markers for AD/other dementias, MCI, or healthy		(67–70)
**Genetic markers**			
APOE genotyping^a^	Identify markers for AD/other dementias, MCI, or healthy	(71, 72)	
Other genes^a^ ADRA2B TPEM2 (R47H) ABCA1	Identify mutation for early AD	(73)	(74, 75)

a *Diagnosis procedures followed in outpatient memory clinics*.

b *Diagnosis procedures followed in day care centers*.

The diagnostic procedures are delivered to PwD, MCI, as well as SCI as detailed below.

### Dementia

#### Criteria

The inclusion criteria for dementia are (a) diagnosis of major neurocognitive impairment of any etiology according to DSM-V criteria (76), (b) MMSE total score ≤ 23, (c) stages 4 and 5 of the disease according to the Global Deterioration Scale (GDS) (10), and (d) absence of anxiety and depression evaluated by the same scales employed for the two previous groups.

#### Neuropsychological Assessment

The most common types of dementia are Alzheimer's disease (AD) and vascular dementia, while frontotemporal dementia (FTD) and Lewy body dementia are less common. The neuropsychological evaluation lasts approximately 2 h, divided into two different face-to-face sessions to obtain the best performance from the participants by reducing the possibility of them getting tired. These tests are administered by a neuropsychologist consisting of screening tools, detection of memory, orientation, and language disorders, and tests of visuospatial ability, attention, executive function, and working memory ability as well as neuropsychiatric symptoms and independent living capacity (Tables 1, 2).

### Mild Cognitive Impairment

#### Criteria

MCI is a transitional state between normal aging and dementia. The inclusion criteria are (a) diagnosis of MCI according to Petersen (77), excluding other pathologies not associated with dementia according to the Diagnostic and Statistical Manual of Mental Disorders, DSM-5 (76), (b) Mini Mental State Examination (MMSE) total score ≥26, (c) stage 3 of the disease according to the GDS, and (d) 1.5 standard deviation (SD) below the normal mean according to age and education in at least one cognitive domain according to the utilized neuropsychological tests.

#### Neuropsychological Tests

In order to identify older adults with MCI, all the psychometric tools used for dementia detection are also administered in MCI using different cutoff scores (Tables 1, 2).

### Subjective Cognitive Impairment Criteria and Tests

To determine SCI, we apply the Subjective Cognitive Decline Questionnaire (SCDQ) (20) and Memory Alternation Test (MAT) (21), which hold excellent reliability and sensitivity for discriminating those with SCI from NCI and MCI patients (Tables 1, 2).

### Laboratory Examinations

#### Regular Blood Test Examination

Blood tests are performed in all patients. Routine blood test includes hematological (complete blood count, hematocrit, and hemoglobulin) and biochemical (glucose, cholesterol, *etc*.), rapid plasma reagin, as well as thyroid-stimulating hormone, and the levels of homocysteine, folic acid, vitamin D, and B12, which are correlated with cognitive impairment. Some patients who participate in clinical trials or clinical research projects, further blood tests or serum tests are performed to identify biological markers or risk genes which are possibly implicated in AD (78, 79).

#### Cerebrospinal Fluid Examination

Cerebrospinal fluid (CSF) samples are taken by lumbar puncture at the L3/L4 or L4/L5 interspace. The samples are stored at 80°C until further examination. CSF-Aβ42 is determined using a sandwich ELISA [INNOTEST β amyloid (1–39, 76, 77) (Lazarou et al. under revision) Innogenetics, Ghent, Belgium-−96 tests]. CSF-total tau levels are determined using the INNOTEST hTau-Antigen sandwich ELISA−96 tests (Innogenetics, Ghent, Belgium) and INNOTEST Phospho TAU protein at threonine-181hyperphosphorylated-tau–96 tests as well. The CSF Fas levels are determined with the human sAPO-1/Fas ELISA (Bender MedSystems, Vienna, Austria).

### Neurophysiological and Neuroimaging Markers

#### Auditory Event-Related Potentials

Auditory event-related potentials (AERPs) are sensitive neurophysiological biomarkers of MCI and AD using a simple discrimination task, the so-called oddball paradigm. In this task, two stimuli are presented in a random series, with one of the two less frequently, i.e., the odd ball. A series of binaural tones at 70 dB sound pressure level with 10-ms rise/fall and 100-ms plateau time is presented to all subjects. The auditory stimuli are presented in a random sequence with target tones of 2,000 Hz occurring 20% of the time and standard tones of 1,000 Hz occurring 80% of the time at a rate of 0.5 Hz. The subject is required to distinguish between the two tones by responding to the target (*e*.*g*., mentally counting) and not responding to the standard (79). The patients must pay attention in distinguishing the tones in order for the examination to be as accurate as possible.

The ERP activity is recorded at the Fz and Pz electrode sites of the 10–20 system using gold-plated electrodes affixed with electrode paste and tape, referred to as linked earlobes at the A1 A2 sites with a forehead ground and impedance at the lowest possible level. For all recordings, the electrode impedances are below 5 kΩ, and they are checked periodically during the recording session. For artifact suppression, an AC filter function was performed. For the purpose of reduced impedance, a special type of paste is used (Elefix Nihon-Kohden, EEG paste Z-401 CE). The AERPs are analyzed by means of Neuropack 4 (Nihon-Kohden, Tokyo).

#### Electroencephalography

Electroencephalography (EEG) activity is acquired in a resting state with a 19-channel Nihon Kohden. Neurofax J 921A EEG system at electrodes Fp1, Fp2, F7, F3, F z, F4, F8, T3, C3, Cz, C4, T4, T5, P3, P z, P4, T6, O1, and O2 of the international 10/20 system [43]. EEG data is sampled at 500 Hz, and the electrode impedance is kept lower than 5 kΩ. The signals are digitized with Neurofax EEG-1200, ver. 01–93. The patients are sitting in a comfortable armchair in a quiet room. They are instructed to remain calm, with their eyes closed, for 5 min and then open their eyes. During the pre-processing state, the EEG signal is bandpass-filtered at 0.5–50 Hz, with a notch filter at 50 Hz. These data are assessed in a qualitative way by neurologists, and quantitative analysis is performed by neurophysiologists and engineers.

Additionally, the HD- EEG EGI 300 Geodesic EEG system (GES 300), which uses a 256-channel Hydro-Cel Geodesic Sensor Net (HCGSN) (EGI Eugene, OR), is also implemented in order to investigate the ERP components and multiple network properties. Using this particular EEG system, it has been revealed that the amplitude of visual N170 ERP can differentiate SCI and MCI from the healthy older adults during a task which assessed the emotional processing of facial stimuli (62). This system is used in participants of clinical studies.

#### MRI

In agreement with radiology departments, brain MRI scans (mostly in 1.5 Tesla) are performed in most patients with cognitive disorders. Each MRI examination consists of the following sequences: T1W (±IV contrast), T2W, FLAIR, DWI/ADC, and 3D T2 FLAIR for volumetry. In some cases, T2^*^/SWI sequences are also included.

### Genetic Markers

#### APOE Genotyping

APOE alleles and different mutations are also tested if patients or family members desire to know about the genetic predisposition. The blood samples used for genotyping are collected in ethylenediaminetetraacetic acid-containing receptacles. DNA is extracted from peripheral blood using the QIAamp Blood DNA purification kit (Qiagen Inc, USA). To determine the APOE genotype, part of the APOE gene (228 bp) containing both polymorphic sites (amino acid positions 112 and 158) is amplified by PCR analysis using the following primers: forward: 5′-GGCACGGCTGTCCAAGGAGCTGCA-3′ and reverse: 5′-GCCCCGGCCTGGTACACTGCCAG-3′, according to the method described in Koutroumani et al. (73).

#### TREM2

TREM 2 examination is performed to patients who desire to know if there is any mutation for the early onset of AD. DNA is extracted from peripheral blood. The mutation of TREM2 (c.140G>A/p.Arg47His) is amplified by PCR analysis. For the PCR, Platinum™ II Hot-Start PCR Master Mix (Thermo Fisher Scientific) was used. Primer sequencing, forward: AACACATGCTGTGCCATCC and reverse: CCCAGGATCCCTGAGAGC, was according to Sanger, using the BigDye terminator v3.1 cycle sequencing kit. Electrophoresis followed in an automated genetic analyzer SeqStudio (Applied Biosystems). The diagnosis is based on comparison with the referral sequence NM_018965.

## Interventions

### Interventions Applied in MCI, SCD, and Healthy Older Adults

Healthy older adults and people with SCI and MCI have the following cognitive and physical trainings. Many of the interventions are published here (80–86).

#### Physical Exercise

The program gives emphasis on fundamental dexterities such as stability, movement, handling, functional ability, and general fitness. Therefore, the program consists of aerobic, strength, flexibility, balance, and mobility exercises two to three times per week. It helps people maintain their good health state, improve their physical and functional abilities and their cognitive function through kinetic stimulations, and additionally sustain or decrease the development of dementia symptoms.

#### Memory and Executive Function Program

This program aims to improve the central executive system of working memory based on Baddeley's model. The main goal is to teach the patient three different coding strategies—double coding, hierarchical processing, and reducing speed—in order to remember a specific number of words presented at the beginning of each session.

#### Cognitive Training by Using Famous Paintings

The program aims to enhance cognitive functions such as attention, visual, and verbal memory and semantic memory and trigger the emotions and imagination of the participants through structured tasks, including famous paintings. They are specifically encouraged to answer questions about art crafts, write a story about the content, and recall significant elements of these paintings at the end of each session. It also gives them the chance to learn about masterpieces of painting and express their emotions toward art.

#### English Language Training

The program aims to improve verbal memory, attention, perception, speech production, comprehension, and learning ability, in general, by learning English as a second language. Specifically, the participants are provided with structured language tasks such as reading and writing as well as listening to simple dialogues between native speakers.

#### Greek Monuments

It is a cognitive training program using the history of ancient monuments. It aims to improve cognitive skills such as attention, memory, perception, creativity, speech, socialization, and orientation during the sessions. It includes audiovisual material about the history of Thessaloniki, while discussion and relevant exercises followed. Actual visits to these monuments followed as a way to improve the social life of older adults, decreasing at the same time any feelings of loneliness.

#### Educational TV

The program aims to enhance attention skills, working memory, and written speech. The participants watch an educational video for 20 min, which include various themes (health, ecology, history, arts, astronomy, philosophy, etc.). After that, the video is divided into smaller sections (to make it easier for the participants to remember), and a therapist asks them about the content. At the same time, the participants make comments about their knowledge in a specific topic while also completing some pencil-and-paper tasks.

#### Computer Exercises

This program aims to improve working memory, attention, language, and visuospatial functions, including several computerized memory exercises. Each participant has a touchscreen and performs the exercises in front of him/her. It does not require knowledge of computers. There are five levels of difficulty in each exercise consisting of the following categories: (1) visual–spatial exercises, (2) speech exercises, (3) numerical exercises, (4) reasonable exercises, and (5) memory exercises.

#### Computer Learning

The goal of the program is to promote the learning process, as well as executive functions, and is mainly delivered to high-level participants. The learning modules are the following: (1) usability and familiarity with a PC—Microsoft Windows XP, (2) Word Processor—Microsoft Office Word 2007, (3) Internet use—Internet Explorer, and (4) using accounts—Microsoft Office Excel 2007.

#### Reality Orientation

The program aims to improve language skills, memory, and attention as well as enhance the quality of life, social skills, and mood. It is comprised of paper-and-pencil tasks. At first, all participants read a specific article and are encouraged to remember it, summarize it, and answer specific questions regarding the content. Thus, they are given tasks focused on language functions, including naming, comprehension, semantic memory, and verbal fluency.

#### RHEA: Cognitive Training by Using Kinetic Instructions

This program enhances the visuospatial abilities, attention, executive function, and language skills *via* the execution of motion instructions. Each session consists of five visuomotor and verbal–kinetic tasks, including visual and verbal kinetic stimuli, respectively. During the tasks, the participants are encouraged to use personal strategies toward executing and completing the tasks.

#### Cognitive Control Training via the Execution of Dual Task

The cognitive control training *via* the execution of dual task has as a basic aim the enhancement of cognitive abilities such as the switch of attention, inhibition, and working memory as well as other attention abilities such as divided and sustained attention. During the program, the participants divide their attention in two tasks using paper and pencil. There are also given stimuli of daily life, such as sounds, puzzles, cards, supermarket products, etc.

#### Attention Training

The attention training aims to enhance attention, executive function, and visual–verbal memory. The program includes teaching of memory strategies and adapt levels of difficulty. Each session consists of 10 cognitive tasks including visual selective attention, working memory and switched attention, shifting of visuospatial attention, and a dual task. The tasks are ecologically valid and derived from activities of daily living (ADL) scale, such as the shopping list and searching in a telephone catalog.

#### Language Intervention

Language intervention aims to enhance the vocabulary, including 10 tasks of semantic expression of language (three tasks), semantic comprehension of language (three tasks), and phonemic expression of language (four tasks), whereas each set of cognitive tasks has three levels of difficulty. The tasks are ecologically valid, as they are derived from ADL scale.

#### Prospective Memory

The program aims to enhance the executive function components, such as working memory and verbal fluency as well as prospective memory (PM). It consists of three tasks in each session: (a) an event-based task (non-focal PM task), (b) a time-based task, and (c) a combination task (the intention should be executed after a specific period of time and if a specific cue appeared). The tasks include occupation with puzzles, watching videos, listening to music, doing handcrafts, reading newspapers, making shopping lists, *etc*.

#### Memory Strategies

##### Cognitive Training of Memory Through Learning of Strategies

The aim of the program is to improve the cognitive and functional performance of the older adult participants with MCI. At the beginning, the participants are taught a variety of internal memory strategies, which include “method of loci,” “keywords,” “visual imagery,” “association,” “categorization,” and so forth. As long as they are taught, the participants are encouraged to use internal strategies in aspects of their everyday life, such as memory for numbers, appointments, events which are going to happen in the near future, and names of individuals and places, so that the transmission of knowledge can succeed.

##### Traveling in Greece

In this program, the participants see pictures of several places in the country. They are asked to answer specific questions in order to practice their working memory function, as well as attention abilities, and improve their verbal fluency performance. Afterwards, all participants present a favorite place among those they have previously seen, and a brief description of the place and personal experiences are followed. Finally, a discussion between all group members takes place.

##### Mental Imagery and Relaxation Techniques

The intervention aims to reduce the anxiety of the participants and help them explore their thoughts and feelings through the interpretation of symbolic mental images. The program includes three relaxation techniques: (a) progressive muscle relaxation, (b) breathing exercises, (c) autogenic relaxation and mental imagery as a cognitive rehabilitative technique. Environmental conditions, including soothing music and fragrant essence, are applied.

### Interventions Applied in People With Dementia

Patients with mild and moderate dementia have the following cognitive and physical training: physical exercise, language intervention, RHEA program, and reality orientation are administered also to PwD based on their physical and cognitive capability.

#### Cognitive Training Using Old Greek Movies

In the current program, parts of Greek movies are presented. One of the main goals of the program is mood improvement because of the pleasant content of these movies. After watching the movie, structured exercises, including memory, attention, and recall, followed. Additionally, the participants are encouraged to share the experiences they may have about the content of the movies.

#### Cognitive Empowerment Using Music Stimuli

The program aims to improve long-term memory, attention, and oral and written language and help them to reduce stress levels and enhance their mood. The participants listen to musical stimuli, and afterward they try to remember facts and experiences related to that song; finally, they perform written exercises about the lyrics.

#### Dance and Drama Therapy

The patients are encouraged to dance and play different roles in order to enhance their executive function abilities, such as planning, step sequence, accuracy, and abstract thinking. This program combines cognitive training *via* psychotherapy techniques, such as dance and drama, and aims to (a) enhance attention, executive function, and verbal and visual memory and (b) deal with the psychological needs of the patients, such as anxiety, depression, apathy, or irritability.

#### Peter Pan: Cognitive Training Through Toy Therapy

This program utilizes toys in order to enhance auditory and visual selective attention, dual-task abilities, working and episodic memory, and language and visuospatial abilities. Executive function and attention abilities are trained using toys, such as dolls, puzzles, plastic letters, plastic animals, and fruits—for example, the participants have to collect plastic fruits and categorize them according to season, color, or size. They were then asked to find words beginning with the first letter of the fruit that they had collected.

#### Psychosocial Activities

Apart from the cognitive training or cognitive stimulation programs applied in PwD and MCI, there are also provided leisure activities and psychotherapeutic sessions for the participants. These activities are as follows: (1) a choir group including PwD and MCI which aims to enhance the mood and self-esteem of a patient, (2) a painting group and an art therapy group which both aim to the expression of feelings and emotions and mental health improvement through painting or other kinds of art, and (3) Gestalt psychotherapy which is applied on patients with MCI. The aim of this psychotherapeutic procedure is the mental health improvement and the reduction of anxiety and mental deficits in general.

### Interventions Applied to Caregivers

There is available published work for caregivers in the some studies (87–91).

#### Psycho-Educational Groups

The aim of the psychoeducational program is to provide information to caregivers regarding the disease and the level of functionality of the patient, in addition to the guidelines for more effective care. Education helps caregivers in making difficult decisions concerning the care and the treatment of their beloved. Caregivers also learn to be flexible in the negotiation of alternative solutions. There is also an online group which satisfies the needs of caregivers who cannot benefit from the face-to-face health support services due to health issues, transportation (due to COVID pandemic-related reasons), or time.

#### Family Psychological Support

Family psychological support aims to help the whole family of people with dementia face and cope with the disease and reduce negative feelings and sense of burden.

#### Support Groups

Support groups aim to help the caregivers to be effective in their role and build up the necessary psychological skills to deal with difficult aspects of the disease and feelings of anger, loneliness, loss, and helplessness. During the support group, caregivers can develop new approaches of interpreting the situation they are dealing with and adapt more realistic targets and more effective pressure and anxiety management strategies.

#### Dyadic Intervention: “Writing Our Couples' Life Book”

The participants are couples, where one partner has been diagnosed with MCI or mild dementia. Based on narrative therapy principles, dyadic intervention helps the couple re-narrate and rewrite their story, including dementia in their common life. Moreover, communication techniques are presented to couples in order to improve their communication skills.

#### Support Group for Grief

It refers to those who experience grief due to the loss of their patient. This group aims to help them accept the reality of loss, manage their emotions, and adapt the new cycle of life.

#### Relaxation Intervention

It aims to reduce the anxiety level and manage psychosomatic symptoms using relaxation techniques and mental imagery which lead to a deep relaxation of the body and mind. Relaxation intervention helps caregivers to develop their well-being and decrease stress levels.

## Further Actions in Contribution to the Field of Dementia

### Development of a Dementia Database: Empedocles Electronic Health Record

Due to the huge amount of data of patients, the creation of a health database was crucial. Thus, an electronic health record (her) system, called Empedocles, was developed in 2016. Software developers, neurologists, psychologists, and other experts worked together to create the database which meets the needs of patients and experts, providing flexibility for different environments and clinical workflows. Empedocles is compliant with the (EU) 2016/679 General Data Protection Regulation by design. The EHR stores the following data on the patients: (1) personal information and demographic characteristics (including geospatial data), (2) medical history, triggers, and risk factors, (3) diagnosis, (4) medication, (5) neurophysiological examination, (6) dental examination, (7) neuropsychological assessment, (8) hematological and biochemical test results, (9) genetic and CSF results, (10) diagnostic neuroimaging test results, (11) perforation results, and (12) assessment of the mental health of caregivers. Currently, Empedocles EHR is hosted on a server at the Aristotle University of Thessaloniki and serves about 132 active users daily. It stores over 5,200 parameters, which can be repeatedly saved in each patient examination. Empedocles has amassed data for over 19,000 patients examined from 1988 till today (visits in memory clinics and DCCs), with more than 45,000 neuropsychological examinations. The database is continually updated and improved following both the requirements of end-users and society. During the COVID-19 pandemic, the functionalities of Empedocles were adapted so that the neuropsychological assessments could be applied from a distance (e.g., via telephone or Skype).

### Clinical Trials

Memory clinic has been participated in several clinical trials to test new drugs for dementia during the last 30 years. The most indicative are provided in Table 4.

**Table 4:** Clinical trials.

**Clinical trial name**	**Main objectives**	**Results**	**Sponsors**
Rivastigmine [Alzheimer's disease (AD) treatment]	Identifying clinical efficacy and safety of rivastigmine for patients with dementia of Alzheimer's type	Rivastigmine was found to be beneficial in patients with mild and moderate AD, mainly in cognitive function and ADL. However, further research should shed light on how to minimize its adverse effects (92)	Department of Clinical Geratology Radcliffe Infirmary, Oxford 6HE, UK/1999–2000
SB-742457 (AD treatment)	A Double-blind, controlled phase II study of a 5-HT6 receptor antagonist, SB-742457, in patients with mild to moderate AD to identify its safety and efficacy	SB-742457 was generally safe and well-tolerated and may be efficacious in AD (93)	GlaxoSmithKline, Uxbridge, Middlesex, UK. Gareth.C.Maher-Edwards@gsk.com/2005–2007
AVA105640 (AD treatment)	A 24-week, double-blind, randomized, parallel-group study to investigate the effects of rosiglitazone (extended release tablets), donepezil, and placebo as monotherapy on cognition and overall clinical response in APOE ε4-stratified subjects with mild to moderate AD	APOE epsilon 4 non-carriers exhibited cognitive and functional improvement in response to rosiglitazone, whereas APOE epsilon 4 allele carriers showed no improvement, and some decline was noted (94)	GlaxoSmithKline R&D Ltd/2006–2008 (+extensions)
Memantine [treatment for dementia, Parkinson's disease (PD), Lewy bodies (LBD)]	An international double-blind study of memantine in patients with dementia, PD and LBD	Memantine seems to improve the global clinical status and behavioral symptoms of patients with mild to moderate LBD and might be an option for the treatment of these patients (95)	Lundbeck/2007–2009
Donepezil (AD treatment)	Efficacy and safety of donepezil. A multicenter double-blind study about the effectiveness and side effects of donepezil in patients with mild to moderate AD (DON-1-97-001)	Donepezil treatment is effective in everyday clinical practice, showing significantly improved cognition, social behavior, and activity in patients with mild to moderate AD (96)	Not available/2008–2010
Galantamine (AD treatment)	Effects and safety of galantamine. A multicenter 2-year, randomized, placebo-controlled study in mild to moderate AD change to galantamine. An international outcome survey in dementia—GAL-ALZ-401 (IOSID)	Galantamine slowed the functional as well as cognitive decline in patients with Alzheimer's disease (97)	Janssen Research Foundation, Beerse, Belgium/2008–2014
Ladder (treatment for AD)	The effect on cognitive performance of Lu AE58054 (idalopirdine), a selective 5-HT6 receptor antagonist, was assessed in donepezil-treated patients with moderate AD	Idalopirdine significantly improved cognition in donepezil-treated patients with AD in moderate stage (98)	Lundbeck/2009–2011
NCT01549834 (treatment for AD)	A randomized, double-blind, placebo-controlled multicenter study investigated the efficacy and safety of ABT-126 in subjects with mild to moderate AD who were taking stable doses of acetylcholinesterase inhibitors (AChEIs)	The efficacy profile of ABT-126 did not warrant further development as add-on therapy to AChEIs to treat mild to moderate Alzheimer's disease (99)	AbbVie Inc., North Chicago, Illinois/2012–2013 (+extensions)
Nilvad (AD treatment)	A European multicenter double-blind placebo-controlled phase III trial of nilvadipine in mild to moderate AD	Pharmacological intervention in AD. The results do not suggest the benefit of nilvadipine as a treatment in a population spanning mild to moderate Alzheimer disease. Future clinical trials of nilvadipine should be restricted to mild and very mild AD patients (100)	FP7/2012–2017
Masitinib (AD treatment)	A multicenter, double-blind, placebo-controlled, randomized, parallel-group phase III study to evaluate the safety and efficacy of masitinib in patients with mild to moderate Alzheimer's disease	The drug appeared to halt cognitive decline, with the treatment group, on average, notching slight improvements on the ADAS-Cog and ADCS-ADL. In addition, fewer patients on the drug, than on placebo, progressed to severe dementia (101)	AB Science/2013–2020
BI 425809 (treatment for AD)	A multicenter, double-blind, parallel-group, randomized controlled study to investigate the efficacy and safety of orally administered BI 425809 during a 12-week treatment period compared to placebo in patients with cognitive impairment due to AD	No results available; ongoing study (102)	Boehringer Ingelheim/2018–2021
Micoil (treatment for MCI)	A randomized clinical trial of Greek high phenolic early harvest extra virgin olive oil in mild cognitive impairment	Long-term intervention with extra virgin olive oil or MP-EVOO was associated with a significant improvement in cognitive function compared to MeDi, independent of the presence of APOE ε4 (103, 104)	GAADRD, World Olive Center for Health (WOCH)-Yanni's Olive Grove Company providing the Early Harvest EVOO, Potidea Chalkidiki, Greece 2018–2020
Olive oil leaves—golden (treatment for MCI)	A single-blind, Randomized Clinical study designed to investigate the Olive Oil Leaves' efficacy to modify treatment and prevention for MCI patients (2019–2021)	No result available; ongoing study (105)	Yanni's Olive Grove Company/2019–2021
Teamentia (treatment for MCI)	Management of mild cognitive impairment patients with Greek mountain tea—Teamentia. A double-blind, randomized clinical study designed to investigate the efficacy of Greek mountain tea as a disease course-modifying treatment for MCI	No result available; ongoing study (106)	Aristotle University of Thessaloniki/2019–2021
NEURO-TTRansform	A study to evaluate the efficacy and safety of AKCEA-TTR-LRx in participants with hereditary transthyretin-mediated amyloid polyneuropathy	No result available; ongoing study (107)	Ionis Pharmaceuticals, Inc. Akcea Therapeutics 2020/2024

### Studies/Projects

Memory clinic and DCCs have also participated in several research studies and international projects the last 30 years. The most indicative are provided in Table 5.

**Table 5:** Research studies and projects.

**Project name, main role, objective**	**Aims**	**Main results**	**Call**
MIRAGE (participant) Genetic markers “A multi-institutional research in Alzheimer's genetic epidemiology”	The goal was to identify genetic and non-genetic risk factors for AD. There were collected data on medical and family history and demographic and lifestyle information, drawing of blood for DNA and analysis, and capturing of data from MRI to evaluate the association between vascular and genetic risk factors and AD in families including Caucasians, African Americans, and Japanese Americans	It has been shown that the E4 variant of apolipoprotein E (APOE) is the strongest AD risk factor identified thus far. Moreover, some vascular risk factors are more prevalent in African American and Japanese American populations than in Caucasians (108)	NIH/2002–2008
ENIR (participant) Biomarkers in MRI “Foresight study for the development of a European neuroimage repository”	The goal was to investigate the scientific needs in relation to the development of a large and shared European multidimensional repository of MRI images of normal brains and brains with different neurodegenerative disorders [AD, Parkinson's disease (PD), etc.] completed by clinical, genetic, and neuropsychological data	Identification of standardized procedures and practical implications and processing and storage of neuroimages by means of a coordinated approach to the setting up of a devoted research infrastructure, making the best use of the already existing repositories, in view of their increased integration toward the development of the future European infrastructure (109)	FP6, 2002–2006
ICTUS (participant) Management of AD “The impact of treatment with anticholinesterase inhibitors (AChE I) on Europeans with AD”	The goals were to find clinical evidence for the global efficacy of AChE I and to develop a picture of the natural history of AD. These outcomes would provide a fundamental insight into the progression and treatment of AD, aiding in the formulation of European guidelines	It was a prospective 2-year observational study which coordinates the centralization of patient data available within the individual centers of the study. The primary outcome measure was a deterioration of one level on the clinical dementia rating scale (110)	FP5/2003–2006
DESCRIPA (participant) *Biomarkers in MCI patients* “DEvelopment of Screening guidelines and clinical CRIteria for Predementia Alzheimer's disease”	The goals were to develop the diagnostic criteria for pre-dementia AD in a clinical setting and the development of screening guidelines for pre-dementia AD in the general population	Screening guidelines and clinical criteria for AD were developed. The clinical criteria were based on a prospective cohort study of non-demented subjects from a memory clinic. The screening guidelines were based on a meta-analysis of prospective population-based cohort studies in Europe (111)	FP5/2003–2010
InnoMed /AddNeuroMed Biomarkers of AD “Innovative medicines in Europe”	AddNeuromed is part of InnoMed, being a cross-European study, designed to find biomarkers or tests for AD. The AD biomarkers are useful for accurate and earlier diagnosis, for prediction of progressing to disease or for more rapid deterioration, and for monitoring the progression of disease	Development of plasma markers. Identification of a range of markers including CFH and A2M, both of which have been independently replicated. Conclusions: (1) collaboration is essential; (2) design is paramount and combining modalities, such as imaging and proteomics, may be informative; (3) animal models are valuable in biomarker research; and (4) plasma markers are feasible (112)	FP6/IMI 2006–2008
EDAR (participant) Biomarkers of AD “Cerebrospinal fluid (CSF) differences in different types of dementia”	Its goal was to develop and validate new biomarkers for AD and to develop an assay for the measurement of beta amyloid oligomers in CSF and plasma	Ultra-sensitive assays were developed to measure oligomers *in vivo*. To validate the assay for beta amyloid oligomers, CSF and plasma were repeatedly collected in subjects with AD, other types of dementia, and MCI and in control subjects (113)	FP6 2007–2010
En-NOHSHS (primary investigator) Management of cognitive impairment via new technologies “An ambient intelligence system for the monitoring, empowerment, and disease evolution prediction for patients with MCI”	Its goal is the exploration and integration of environmental factors as well as the effect of activities of daily living with medical and biological factors in order to monitor and predict AD/MCI disease progression and evolution	The system used IT-based tools (including also 3D gaming environments) in order to address specific cognitive and physical/motor parameters and ADL factors within AD and MCI domain to improve their diagnosis, evaluating their variations along the progress of the AD and its different steps and supporting the stimulation/training of the patient affected by the disease (114)	“COLLABORATION” 2009–2011
LLM (participant) Management of AD *via* new technologies “Long-lasting memories”	Its goal is the development of an integrated ICT platform which combines state-of-the-art cognitive exercises with physical activity in the framework of an advanced ambient assisted living environment	By combining cognitive exercises and physical activity, LLM delivers an effective countermeasure against age-related cognitive decline, thus actively improving the quality of life of the elderly and significantly prolonging the time that they can remain independent at home while respecting ethical and legal boundaries (115)	FP6 2009–2012
PHARMACOG (participant) Multiple biomarkers of AD “Prediction of cognitive properties of new drug candidates for neurodegenerative diseases in early clinical development”	Its goal is to provide the tools needed to define more precisely the potential of a drug candidate, reduce the development time of new medicines, and thus accelerate the approvals of promising new medicines	It developed a matrix of biomarkers which can be used to study the effect of a drug candidate both in animals and humans and has the potential to predict the success of future drugs more accurately in the early stages of drug development. It also found a better way to stratify patients with early signs of AD, which may lead to more definitive clinical trials. At least one biotech company is already using the results of Pharma-Cog to test a promising new drug candidate (116)	IMI/2010–2015
Dem@Care (participant) Management of cognitive impairment *via* new technologies “Dementia ambient care multi-sensing monitoring for intelligent remote management and decision support”	Using biosensors, it contributes to the timely diagnosis, assessment, maintenance, and promotion of self-independence of PwD by deepening the understanding of how the disease affects their everyday life and behavior	Positive impact regarding the implementation of a multi-parametric closed-loop remote management solution that affords adaptive feedback to the PwD while including clinicians into the remote follow-up, enabling them to maintain a comprehensive view of the health status and progress of the affected person (117)	FP7/ 2011–2015
ASPAD (principal investigator) Management of MCI patients and support care givers *via* new technologies “Exploring the potential of programming tasks to benefit patients with MCI”	Its goals were the computerized exercises and support groups through the Internet. Exploration of the potential of robot programming tasks to benefit patients with MCI when implemented as a form of cognitive training with the use of user-friendly tangible interface	Available evidence encourages further investigation of the impact of programming tasks on MCI patients, as a cognitive training and assessment tool, in relation to important mental skills (such as analysis and planning) and cognitive processes such as attention (118)	“EXCELLENCE, ESPA”-2012
BIOMARK-APD (participant) Biomarkers for AD and PD	Its goal was to improve the clinical use of body fluid markers for the diagnosis and prognosis of AD and PD. The objective was to standardize the assessment of existing assays and to validate novel fluid biomarkers for AD and PD	(1) New and better assays to test the new and better biomarker candidates; (2) certified reference materials that can be used to harmonize assays that are used to measure the different biomarkers were developed. A virtual biobank with 8,600 subjects and varying diagnoses from 21 local biobanks was also created. A website has been launched to enable sample requests from the central biobank and virtual biobank and standardized assays (119, 120)	JPND/2012–2016
CBP (participant) Diagnosis of cognitive impairment *via* new technologies (EEG) “Cognitive brain signal processing lab”	Its goal was to advance the state of the art in vector field tomography (VFT) by exploiting the new methodology in 2D and extending its theory to 3D. Subsequently, the goal was to apply 3D-VFT to high-density EEG data to solve the inverse EEG problem and determine the active states of the brain to understand the cognitive process	The project not only advanced the state of the art in VFT on its own but also served as a tool to understanding cognitive processes in the brain and, in particular, cognitive vision through different experimental scenarios and among different experimental groups^a^	EXCELLENCE, ESPA/2013–2015
HARC (participant) Education of European physicians “Healthy aging research centers”	Its goal was the development of research focusing on major areas relevant to active and healthy aging: novel approaches to improve well-being in the elderly; pathogenesis and prevention of neurodegenerative, respiratory, cardiovascular and kidney diseases; and molecular basis of aging	It allowed for the enhanced integration of research consortium and extended collaboration with international partners, resulting in achieving significant progress in research (121)	FP7/2013–2016
ARCHIMIDIS (participant) “ MCI and diabetes mellitus (DM)”	Its goal was to assess the cognitive function of DM and MCI patients with neurophysiological and neuropsychological measures and seek for possible correlations	No difference in the AERP characteristics and the neuropsychological performance between the groups. The higher cognitive functions of DM patients as assessed with ERPs and neuropsychological tests are affected in a similar way with that of MCI patients; this supports the existence of common pathophysiological mechanisms between the two diseases (122)	ESPA/2014–2016
ehcoBUTLER (participant) Management of MCI patients *via* new technologies “A global ecosystem for the independent and healthy living of elder people with mild cognitive impairment”	It is a multidisciplinary study approach, designed to test the socio-economic benefits from the deployment of an innovative and user-led ICT platform with both leisure and care apps to promote the independence and quality of life and good health of elderly people.	The outcomes of this ongoing project will determine any relevant changes in cognition, mood, quality of life, activities of daily living, and quality of patient–carer relationship after 4 months and 1-year follow up of intervention in a cross-sectional group comparison (123)	Horizon 2020/2015–2016, interrupted and started again 2019–2022
Serious Games-AD GAMING (participant) Management of cognitive impairment via new technologies “Development of a training program for the improvement of the quality of life of PwD”	Its aim is to improve the technological skills of PwD, their families and caregivers, thus allowing them to use Serious Games (SGs) with the purpose of improving their quality of life	The practitioners and care partners found the SG training platform useful and were excited about the prospect of using it to support the well-being of PwD^b^	ERASMUS +/2016–2018
ALTOIDA (participant) Management of cognitive impairment *via* new technologies “A revolutionary assessment test for cognitive impairment. Cognitive biomarker for AD”	It was designed to evaluate the performance of the ALTOIDA™ System as a tool to assist physicians in diagnosing AD in real-world clinical settings. It tests the functional and cognitive aptitude of a patient, via a self-learning (ML) algorithm	Accurate assessment in <10 min; higly validated cognitive assessment for patients over 62 years old. Report on brain patterns of certain neurologic conditions such as cognitive impairment and AD. These data should only be used as additional information to add to the diagnostic impression of the primary physician^c^ (124)	Altoida 2016–2020
iCONNECT Education of students *via* new technologies “Intergenerational CONtact between studeNts and people with dementia through CreaTive education”	Its goal is the development of innovative practices in supporting the social engagement of higher educational institutions in promoting via intercultural and intergenerational support the social inclusion of older people with dementia	The only published output is regarding the needs analysis^d^	Erasmus+/2017–2020
RECage (participant) Management of BPSDs “Special medical care unit for patients with BPSD (SCU-B)”	Its goal is to assess the short- and long-term effectiveness of the SCU for people with dementia and BPSD toward alleviating BPSD and improving the quality of life of patients with dementia and their caregivers	This is a 3-year ongoing prospective study where 500 persons will be enrolled (125)	Horizon/2018–2023
STORY2REMEMBER (participant) Education of healthcare professionals “Drama and storytelling in dementia care”	Its goal is to improve the quality of life of both PwD and their caregivers and to improve the skills of healthcare professionals skills through training with methodology of drama and storytelling	A training handbook was produced as a final product for professionals to deliver the experiential workshops to PwD based on storytelling and creative drama techniques. The program had a positive impact to the well-being of PwD and their caregivers as well as positively benefiting professionals working in dementia care settings (126)	Erasmus+/2018–2021
BRIDGE (participant) Management of AD *via* new technologies “An intergenerational approach using serious games for PwD”	Its goal is to develop a set of prototypes Serious Games (physical, digital, and phygital) acting on cognitive and behavioral symptoms of dementia, involving also younger and older people	Prototype games entitled: next destination, flea market, find the word, bird-watching, emotions, the directors, blooming flowers, speciatite—tested during a series of workshops. The web platform containing massive open online courses on the methodology of the game-creation workshops and the final eight selected serious games is the final result^e,f^	Erasmus+/2018–2021
E.LSoM.C.I (participant) Management of MCI *via* teaching “A European intervention with English lessons using songs for people with MCI”	Its goal is to teach English language to people with MCI using songs as the main tool of the teaching process. It is based on innovative teaching approaches, places great emphasis on verbal communication, creates a positive environment in class, reduces stress, and encourages learners to learn step by step naturally and pleasantly	It is an ongoing program; thus, so far it has prepared a methodological guide that provides trainers with a lesson plan for each lesson and many teaching aids, such as songs, images, flashcards, Powerpoint presentations, and videos as well as interactive activities such as role playing, chain drills, and games	Erasmus+/2020–2023
RADAR-AD (participant) Management of AD *via* new technologies “Remote assessment of disease and relapse—Alzheimer's disease”	Its main aim is to explore how mobile and digital technologies—such as smart phones, wearables, and home-based sensors—can be used in AD assessment and care and to measure disability progression associated with AD	It is an ongoing program with no results published yet. Technological techniques might help to detect AD earlier. Mobile technology also allows a more personalized approach to AD treatment and care so that PwD can live independently for longer. It will also identify “digital biomarkers” (electronic signals that give information about a person's health status) for AD, creating new perspectives for the development of treatments against this progressive condition (127, 128)	Horizon 2020/IMI-EFPIA/2019–2022
VRADA (participant) Management of MCI *via* new technologies and physical exercise “A virtual reality (VR) app for physical and cognitive training of older people with MCI: mixed methods feasibility study”	Its goal is to design and test the acceptability, usability, and tolerability of an immersive VR platform that allows older people with MCI symptoms to simultaneously practice physical and cognitive skills on a dual task	The findings suggest that VRADA is an acceptable, usable, and tolerable system for physical and cognitive training of older people with MCI and university students. Randomized controlled trial studies are needed to assess the efficacy of VRADA as a tool to promote physical and cognitive health in patients with MCI. The program is ongoing^g^	ESPA/2018–2021

a *http://cbp.iti.gr/*.

b *https://adgaming.ibv.org/en/training-content/*.

c *https://altoida.com/*.

d *https://www.iconnectdementia.eu/*.

e *https://projectbridge.eu/the-serious-game/*.

f *http://bridgecourses.uowm.gr/*.

g *http://ikee.lib.auth.gr/record/329332*.

## Conclusions

All the above-mentioned efforts have the following as targets:

- To provide a protocol of a holistic evaluation of cognitive status through clinical examination, an extended neuropsychological assessment, and biomarkers like blood tests, CSF, genetic tests, and MRI scans.
- To detect cognitive disorder as early as possible and carry out a differential diagnostic procedure to identify their etiologies.
- Plan the future care and provide advice to patients and their caregivers with respect to medical, psychological, legal, ethical, and social issues.
- Provide direct support to patients and caregivers by means of counseling, discussions with caregivers, and therapeutically oriented workgroups (e.g., memory training groups)
- Support families either at our day centers or at their homes
- Contribution to the dementia research and clinical field through funded projects and a plethora of studies conducted in DCC's and Outpatient Memory Clinics.

## Author Contributions

All authors listed have made a substantial, direct and intellectual contribution to the work, and approved it for publication.

## Conflict of Interest

The authors declare that the research was conducted in the absence of any commercial or financial relationships that could be construed as a potential conflict of interest.

## Publisher's Note

All claims expressed in this article are solely those of the authors and do not necessarily represent those of their affiliated organizations, or those of the publisher, the editors and the reviewers. Any product that may be evaluated in this article, or claim that may be made by its manufacturer, is not guaranteed or endorsed by the publisher.
